# Factors associated with death in confirmed cases of COVID-19 in the state of Rio de Janeiro

**DOI:** 10.1186/s12879-021-06384-1

**Published:** 2021-07-16

**Authors:** Marcella Cini Oliveira, Tatiana de Araujo Eleuterio, Allan Bruno de Andrade Corrêa, Lucas Dalsenter Romano da Silva, Renata Coelho Rodrigues, Bruna Andrade de Oliveira, Marlos Melo Martins, Carlos Eduardo Raymundo, Roberto de Andrade Medronho

**Affiliations:** 1 Faculdade de Medicina, Universidade Federal do Rio de Janeiro, Rio de Janeiro, Brasil; 2 Instituto de Estudos em Saúde Pública / Faculdade de Medicina, Universidade Federal do Rio de Janeiro, Rio de Janeiro, Brasil; 3 Faculdade de Enfermagem, Universidade do Estado do Rio de Janeiro, Rio de Janeiro, Brasil; 4 Instituto de Física, Universidade Federal do Rio de Janeiro, Rio de Janeiro, Brasil; 5 Departamento de Medicina Preventiva, Instituto de Estudos em Saúde Pública / Faculdade de Medicina, Universidade Federal do Rio de Janeiro, Rio de Janeiro, Brasil; 6 Department of Child Neurology, Martagão Gesteira Institute of Childcare and Pediatrics, Federal University of Rio de Janeiro, Rio de Janeiro, Brazil

**Keywords:** COVID-19, SARS-CoV-2, XGBoost, Machine learning, Pandemic, Coronavirus infection, Coronavirus death

## Abstract

**Background:**

COVID-19 can occur asymptomatically, as influenza-like illness, or as more severe forms, which characterize severe acute respiratory syndrome (SARS). Its mortality rate is higher in individuals over 80 years of age and in people with comorbidities, so these constitute the risk group for severe forms of the disease. We analyzed the factors associated with death in confirmed cases of COVID-19 in the state of Rio de Janeiro. This cross-sectional study evaluated the association between individual demographic, clinical, and epidemiological variables and the outcome (death) using data from the Unified Health System information systems.

**Methods:**

We used the extreme boosting gradient (XGBoost) model to analyze the data, which uses decision trees weighted by the estimation difficulty. To evaluate the relevance of each independent variable, we used the SHapley Additive exPlanations (SHAP) metric. From the probabilities generated by the XGBoost model, we transformed the data to the logarithm of odds to estimate the odds ratio for each independent variable.

**Results:**

This study showed that older individuals of black race/skin color with heart disease or diabetes who had dyspnea or fever were more likely to die.

**Conclusions:**

The early identification of patients who may progress to a more severe form of the disease can help improve the clinical management of patients with COVID-19 and is thus essential to reduce the lethality of the disease.

**Supplementary Information:**

The online version contains supplementary material available at 10.1186/s12879-021-06384-1.

## Background

In December 2019, cases of pneumonia of unknown cause occurred in Wuhan, Hubei Province, China. On January 7, 2020, a new beta-coronavirus called severe acute respiratory syndrome coronavirus 2 (SARS-CoV-2) was identified [1–3]. Like severe acute respiratory syndrome coronavirus and Middle East respiratory syndrome coronavirus, SARS-CoV-2 causes a lower respiratory infection called coronavirus disease 2019 (COVID-19). The World Health Organization (WHO) declared the disease a public health emergency of international concern on January 30, 2020. On February 3, 2020, Brazil announced the disease as a public health emergency of national concern [4], and on February 25, 2020, the country confirmed its first case [5].

On June 14, 2021, the world had 175,686,814 confirmed cases and 3,803,592 deaths, and Brazil had 17,452,612 confirmed cases and 488,228 deaths [6]. COVID-19 can occur asymptomatically, as influenza-like illness, or as more severe forms, which characterize severe acute respiratory syndrome (SARS). The most common clinical manifestations include fever, dry cough, diarrhea, vomiting, and muscle pain or fatigue [7, 8]. The average incubation period is 5.2 days [9]. Transmission can occur by droplets and aerosols or by contact with contaminated objects [10].

Its mortality rate is higher in individuals over 80 years of age and in people with comorbidities, so these constitute the risk group for severe forms of the disease [11]. The gold standard for laboratory confirmation of SARS-CoV-2 infection is reverse transcription-polymerase chain reaction (RT-PCR). The performance of mass RT-PCR testing, contact tracing, and isolation of positive individuals is essential to control the spread of the virus [12–15].

This study aims to analyze the factors associated with death in confirmed cases of COVID-19 in the state of Rio de Janeiro and considers the importance of defining these factors, the objective of several studies in the literature, for better case management [16–21].

## Methods

### Design

The design was a cross-sectional study evaluating the association between individual demographic, clinical, and epidemiological predictor variables and the outcome (death) among confirmed cases of COVID-19 in the state of Rio de Janeiro using data gathered from the Unified Health System (SUS) information systems.

### Data collection

We gathered the confirmed influenza-like illness cases caused by COVID-19 from the e-SUS NOTIFICA system of the Ministry of Health. We obtained the confirmed SARS cases by COVID-19 from the Flu Epidemiological Surveillance Information System (*SIVEP-Gripe*) of the Ministry of Health. We linked these two databases. The consistency analysis removed any duplicates from the resulting database. Next, we linked this database with the Mortality Information System database of the Ministry of Health. The study covered the period from March 5 (the first confirmed case in the state of Rio de Janeiro) to September 15, 2020. During this period, the state of Rio de Janeiro confirmed 243,509 cases of COVID-19, of which 178,231 (73.2%) were of influenza-like illness (ILI) and 65,278 (26.8%) were of SARS. All cases included in this study met the ILI and SARS criteria defined by the Ministry of Health [22].

The individual predictor variables selected covered demographic, clinical, and epidemiological characteristics (Table 1).

**Table 1:** Individual variables selected

**Demographic**	Age
	Sex
	Race/skin color, self-reported
	Municipality of residence
**Clinical**	Fever
	Cough
	Sneezing
	Runny nose
	Sore throat
	Dyspnea
	Loss of smell
	Loss of taste
	Headache
	Muscle pain
	Diarrhea
**Epidemiological**	Chronic respiratory disease
	Chronic kidney disease
	Diabetes
	Immunodeficiency/immunosuppression
	Heart disease

The immunodeficiency/immunosuppression, considered as a risk factor/ comorbidity both in the notification and investigation of ILI and SARS cases, is defined as any cause of suppression or deficiency of the immune system associated with medications (corticosteroids, chemotherapy, TNF-alpha inhibitors) or pathologies (neoplasms, HIV/AIDS, among others).

### Data analysis

We used Microsoft Excel 365 software to handle the database, while we performed the statistical analyses with the software R × 64 4.0.0 through RStudio Desktop and Python 3.8.5.

In the descriptive analysis of the data, we categorized the quantitative predictor variable age according to age groups (in years): 0 to 9, 10 to 19, 20 to 29, 30 to 39, 40 to 49, 50 to 59, 60 to 69, 70 to 79, and 80 years or older. For the modeling, we did not categorize patient age.

The bivariate descriptive analysis used bar graphs, pie charts, or box plots for the numerical predictor variable (age) and contingency tables for categorical predictor variables. We used Pearson’s chi-squared test to analyze categorical predictor variables and the Wilcoxon test for the numerical predictor variables, both with a level of significance of 5%.

We checked the database for missing data. We kept variables considered epidemiologically relevant but with a high percentage of missing data in the analysis, creating a category named “missing” for each predictor variable. They were “kidney diseases”, “immunodeficiency/immunosuppression”, “diabetes”, “heart disease”, “dyspnea”, “cough”, “fever”, “headache”, “sneezing”, “odynophagia”, “muscle pain”, “diarrhea”, “loss of smell”, “loss of taste”, and “runny nose”.

Due to a large number of categories of the predictor variable “municipality of residence” (91 municipalities), we grouped the residences into regions defined by the state government of Rio de Janeiro [23]. The exception was the city of Rio de Janeiro, which has more than one-third of the state’s population and more than half of the deaths, so we analyzed it as an individual category, called “Metropolitan Region I - capital”.

Then, through the evaluation with several machine learning algorithms, we applied an Extreme Boosting Gradient (XGBoost), as it showed the best performance for the dataset. To meet the assumption of balanced data for a good performance of machine learning models, we balanced the predicted variable (outcome) using the synthetic minority oversampling technique (SMOTE) [24] and edited nearest neighbor (ENN) [25] techniques. The XGBoost model uses decision trees weighted by the estimation difficulty [26]. Compared to machine learning models in general, the advantages of this model are the better generalization of the results, the possibility of visualizing all decision trees, and the reduction of the bias and variance of a single tree.

To evaluate the relevance of each predictor variable, we used the SHapley Additive exPlanations (SHAP) metric [27], which can take negative or positive values, and the closer it is to 0, the less the predictor variable influences the predicted variable. The results show the mean of the modules of the importance of each predictor variable.

Also, we used sensitivity, specificity, accuracy, positive predictive value, negative predictive value, Youden’s index, receiver operating characteristic (ROC) curves, and Matthews’ correlation coefficient (MCC) to evaluate the goodness of fit [28].

From the probabilities generated by the XGBoost model, we transformed the data to the logarithm of odds, where odds is the ratio between the probability of death and non-death attributed to the categories of each predictor variable for each individual. We used the logarithm of odds to estimate the odds ratio, according to the formula below:

$${Odds\ ratio}_i\left({OR}_i\right)=\frac{e^{mean\left(\log \left({odds}_{int}\right)\right)}}{e^{mean\left(\log \left({odds}_{ref}\right)\right)}}$$

Where:

$$\mathit{\operatorname{int}}=\mathrm{Category}\ \mathrm{of}\ \mathrm{interest}\ \mathrm{of}\ \mathrm{the}\ \mathrm{predictor}\ \mathrm{variable}$$

$$ref=\mathrm{Category}\ \mathrm{of}\ \mathrm{reference}\ \mathrm{of}\ \mathrm{the}\ \mathrm{predictor}\ \mathrm{variable}$$

For the numerical variable age, we used the categories of interest described earlier in this section to obtain the summarized results.

We calculated the confidence interval according to Wayne W. LaMorte [29] and estimated the total number of individuals by category and outcome using the following formula:

$$95\% confidence\ interval={e}^{\left(\log \left({OR}_i\right)\pm \left[1,96\times SE\left(\log \left({OR}_i\right)\right)\right]\right)}$$

Where:

$$\mathrm{SE}\left(\log \left({\mathrm{OR}}_{\mathrm{i}}\right)\right)=\sqrt{\frac{1}{{\mathrm{p}}_{\mathrm{i}\mathrm{nt}}\times {\mathrm{n}}_{\mathrm{i}\mathrm{nt}}}+\frac{1}{\left(1-{\mathrm{p}}_{\mathrm{i}\mathrm{nt}}\right)\times {\mathrm{n}}_{\mathrm{i}\mathrm{nt}}}+\frac{1}{{\mathrm{p}}_{\mathrm{ref}}\times {\mathrm{n}}_{\mathrm{ref}}}+\frac{1}{\left(1-{\mathrm{p}}_{\mathrm{ref}}\right)\times {\mathrm{n}}_{\mathrm{ref}}}}$$

$$p= estimated\ probability\ of\ death\ for\ the\ category$$

$$n= number\ of\ in dividuals\ in\ the\ category$$

The authors used the odds ratio confidence interval to calculate its *p*-value, according to the formula present in Altman [30] for proportions. The significance level considered was 5%.

## Results

Between March 10 and September 15, 2020, the state of Rio de Janeiro reported 243,509 confirmed cases of COVID-19, of which 37.3% were residents of the municipality of Rio de Janeiro. Of the total number of cases, 178,231 (73.2%) were classified as ILI, and 65,278 (26.8%) were classified as SARS.

Table 2 shows the incidence, mortality, and lethality of confirmed cases of COVID-19 by age group. The disease incidence was higher in the age group 80 years and older, followed by 40 to 49 years and 30 to 39 years. Mortality and lethality increased with increasing age, except in children younger than 10 years.

**Table 2:** Distribution of incidence, mortality, and lethality by age group

Age group^a^	Deaths	Cases	Population^b^	Incidence^c^	Mortality^c^	Lethality
0 to 9 years	60	5105	2,224,713	229.47	2.70	1.18
10 to 19 years	34	7478	2,193,282	340.95	1.55	0.45
20 to 29 years	210	30,426	2,638,726	1153.06	7.96	0.69
30 to 39 years	603	53,654	2,692,631	1992.62	22.39	1.12
40 to 49 years	1400	51,913	2,476,855	2095.92	56.52	2.70
50 to 59 years	2526	40,729	2,146,886	1897.12	117.66	6.20
60 to 69 years	4154	27,034	1,645,437	1642.97	252.46	15.37
70 to 79 years	4425	15,273	888,202	1719.54	498.20	28.97
80 years and older	4622	10,812	459,457	2353.21	1005.97	42.75
**Total**	**18,034**	**242,424**	**17,366,189**	**1395.95**	**103.85**	**7.44**

Note: confirmed cases of COVID-19, state of Rio de Janeiro, March–September 2020

^a^ Cases with available age information

^b^ Population estimated by the IBGE for 2020

^c^ per 100,000 inhabitants

Table 3 shows the predictor variables’ distribution as a function of disease severity (ILI and SARS). There was a higher frequency of cases among the age group of 40–49 years (21.3%), among females (52.4%), and among brown individuals (32.9%), followed by white individuals (32.6%). The most frequent signs and symptoms were cough, fever, dyspnea, and odynophagia. The most prevalent comorbidities were cardiovascular disease and diabetes mellitus. Regarding disease evolution, the lethality was 7.4%.

**Table 3:** Distribution of confirmed cases of ILI and SARS, according to the individual variables selected

Variables	Overall		ILI		SARS		Statistic parameter	***P***-value
	n	%	n	%	n	%	Chi-square	
**Age group**							18,456	0.000
0–9 years	5105	2.1%	4369	2.5%	736	1.1%	–	–
10–19 years	7478	3.1%	6442	3.6%	1036	1.6%	–	–
20–29 years	30,426	12.5%	25,014	14.0%	5412	8.3%	–	–
30–39 years	53,654	22.0%	43,466	24.4%	10,188	15.6%	–	–
40–49 years	51,913	21.3%	40,163	22.5%	11,750	18.0%	–	–
50–59 years	40,729	16.7%	29,536	16.6%	11,193	17.1%	–	–
60–69 years	27,034	11.1%	16,751	9.4%	10,283	15.8%	–	–
70–79 years	15,273	6.3%	7356	4.1%	7917	12.1%	–	–
80 years or older	10,812	4.5%	4055	2.3%	6757	10.4%	–	–
No Information	1085	40.0%	1079	60.0%	6	0.0%	–	–
**Sex**							7	0.008
Female	127,677	52.4%	93,740	52.6%	33,937	52.0%	–	–
Male	115,832	47.6%	84,491	47.4%	31,341	48.0%	–	–
**Race/skin color**							723	0.000
Brown	80,162	32.9%	60,806	34.1%	19,356	29.7%	–	–
Asian	11,875	4.9%	9808	5.5%	2067	3.2%	–	–
White	79,495	32.6%	58,986	33.1%	20,509	31.4%	–	–
Indigenous	491	0.2%	441	0.2%	50	0.1%	–	–
Black	15,802	6.5%	11,007	6.2%	4795	7.3%	–	–
Not reported	55,684	22.9%	37,183	20.9%	18,501	28.3%	–	–
**Presence of signs and symptoms**							–	–
Fever	93,102	38.3%	53,721	30.1%	39,381	60.3%	21,309	0.000
Cough	108,434	44.5%	65,565	36.8%	42,869	65.7%	19,156	0.000
Headache	33,020	13.6%	24,667	13.8%	8353	12.8%	7	0.007
Sneezing	1636	0.7%	1249	0.7%	387	0.6%	4	0.040
Odynophagia	42,797	17.6%	28,276	15.9%	14,521	22.2%	1749	0.000
Dyspnea	52,376	21.5%	0	0.0%	52,376	80.2%	191,664	0.000
Runny nose	18,139	7.4%	13,931	7.8%	4208	6.4%	75	0.000
Diarrhea	12,801	5.3%	9059	5.1%	3742	5.7%	78	0.000
Muscle pain	23,015	9.5%	16,163	9.1%	6852	10.5%	199	0.000
Loss of smell	21,764	8.9%	16,272	9.1%	5492	8.4%	6	0.020
Loss of taste	15,478	6.4%	11,826	6.6%	3652	5.6%	46	0.000
**Presence of comorbidities**							–	–
Cardiovascular disease	25,845	10.6%	10,296	5.8%	15,549	23.8%	27,410	0.000
Diabetes mellitus	15,500	6.4%	5333	3.0%	10,167	15.6%	21,888	0.000
Chronic kidney disease	2203	0.9%	530	0.3%	1673	2.6%	4930	0.000
Chronic respiratory disease	6318	2.6%	3036	1.7%	3282	5.0%	2079	0.000
Immunodeficiency/ immunosuppression	2527	1.0%	1226	0.7%	1301	2.0%	1897	0.000
**Evolution**							30,186	0.000
Death	18,076	7.4%	2097	1.2%	15,979	24.5%	–	–
Non-death	225,433	92.6%	176,134	98.8%	49,299	75.5%	–	–
**Total**	**243,509**	**100.0%**	**178,231**	**73.19%**	**65,278**	**26.81%**	–	–

Note: confirmed cases of COVID-19, state of Rio de Janeiro, March–September 2020

ILI was more frequent among younger individuals, the most common age group being 30–39 years (24.4%); the median age at ILI diagnosis was 41 years, and it was more frequent among females (52.6%) and brown individuals (34.1%), followed by white individuals (33.1%). The most frequent signs and symptoms were cough, fever, and odynophagia. The frequency of comorbidities was 5.8% for cardiovascular disease and 3.0% for diabetes mellitus. Only 1.2% of ILI cases progressed to death.

SARS was more frequent in individuals older than 60 years (38.3%); the median age was 53. Women had the highest frequency (52.0%), but concerning race/color, white individuals had the highest frequency (31.4%), followed by brown individuals (29.7%). Dyspnea (80.2%), cough (65.7%), and fever (60.3%) were more frequent than in cases of ILI. The rate of associated comorbidities was also higher than that in ILI, with 23.8% cardiovascular disease and 15.6% diabetes mellitus. The lethality observed in cases of SARS was 24.5%.

The statistics of Pearson’s chi-square tests and respective *p*-values ​​are listed in Tables 3 and 4. *P*-values ​​under 0.05 indicate a rejection of the hypothesis of independence between the observed variables. All predictor variables were significantly different between ILI and SARS cases.

**Table 4:** Distribution of COVID-19 outcomes according to the individual variables selected

Variables	Death		Non-death		Statistic parameter	***P***-value
	n	%	n	%	Chi-square	
**Age group**					40,056	0.000
0–9 years	60	0,3%	5045	2.2%	–	–
10–19 years	34	0,2%	7444	3.3%	–	–
20–29 years	210	1,2%	30,216	13.4%	–	–
30–39 years	603	3,3%	53,051	23.5%	–	–
40–49 years	1400	7,7%	50,513	22.4%	–	–
50–59 years	2526	14,0%	38,203	16.9%	–	–
60–69 years	4154	23,0%	22,880	10.1%	–	–
70–79 years	4425	24,5%	10,848	4.8%	–	–
80 years or older	4622	25,6%	6190	2.7%	–	–
**Sex**					521	0.000
Female	8003	44,3%	119,674	53.1%	–	–
Male	10,073	55,7%	105,759	46.9%	–	–
**Race/color**					794	0.000
Brown	4480	24,8%	75,682	33.6%	–	–
Asian	182	1,0%	11,693	5.2%	–	–
White	5041	27,9%	74,454	33.0%	–	–
Indigenous	11	0,1%	480	0.2%	–	–
Black	1479	8,2%	14,323	6.4%	–	–
Missing	6883	38,08%	48,801	21.6%	–	–
**Presence of signs and symptoms**					–	–
Fever	8723	48,3%	84,379	37.4%	830	0.000
Cough	8908	49,3%	99,526	44.1%	178	0.000
Headache	503	2,8%	32,517	14.4%	1934	0.000
Sneezing	10	0,1%	1626	0.7%	110	0.000
Odynophagia	1560	8,6%	41,237	18.3%	1078	0.000
Dyspnea	10,204	56,5%	42,172	18.7%	14,118	0.000
Runny nose	233	1,3%	17,906	7.9%	1074	0.000
Diarrhea	143	0,8%	12,658	5.6%	781	0.000
Muscle pain	803	4,4%	22,212	9.9%	571	0.000
Loss of smell	194	1,1%	21,570	9.6%	1483	0.000
Loss of taste	99	0,5%	15,379	6.8%	1106	0.000
**Presence of comorbidities**					–	–
Cardiovascular disease	6163	34,1%	19,682	8.7%	21,646	0.000
Diabetes mellitus	4341	24,0%	11,159	5.0%	26,588	0.000
Chronic kidney disease	923	5,1%	1280	0.6%	11,461	0.000
Chronic respiratory disease	845	4,7%	5473	2.4%	346	0.000
Immunodeficiency/ immunosuppression	464	2,6%	2063	0.9%	2153	0.000
**Total**	**18,076**	**7,4%**	**225,433**	**92.6%**	–	–

Note: confirmed cases of COVID-19, state of Rio de Janeiro, March–September 2020

Table 4 shows the relationship between the predictor variables and the outcome (death). There was a higher proportion of elderly age (over 60 years old), male sex, and black race/skin color among deaths than among non-deaths. The signs and symptoms that stood out were dyspnea, fever, and cough. Dyspnea was present in 56.5% of cases of death and in 18.7% of non-deaths. Fever was present in 48.3% of the cases of death and in 37.4% of the cases of non-death. Cough was present in 49.3% of cases of death and 44.1% of cases of non-death. The most common comorbidities in both groups were cardiovascular disease, at 34.1% in cases of death and 8.7% in cases of non-death, and diabetes mellitus, at 24.0% in cases of death and 5.0% in cases of non-death.

The number of missing data of predictor variables were as follows: 1085 (0.4%) for age, 3506 (1.4%) for signs and symptoms, 396 (0.2%) for chronic respiratory disease, 55,684 (22.9%) for skin color/race, 19,006 (7.8%) for cardiovascular disease, 21,701 (8.9%) for diabetes mellitus, 25,870 (10.6%) for chronic kidney disease, 26,181 (10.8%) for immunodeficiency/immunosuppression, and 273 (0.1%) for municipality of residence.

There was a statistically significant association between the outcome and the predictor variables listed in Table 4.

**Fig. 1:**
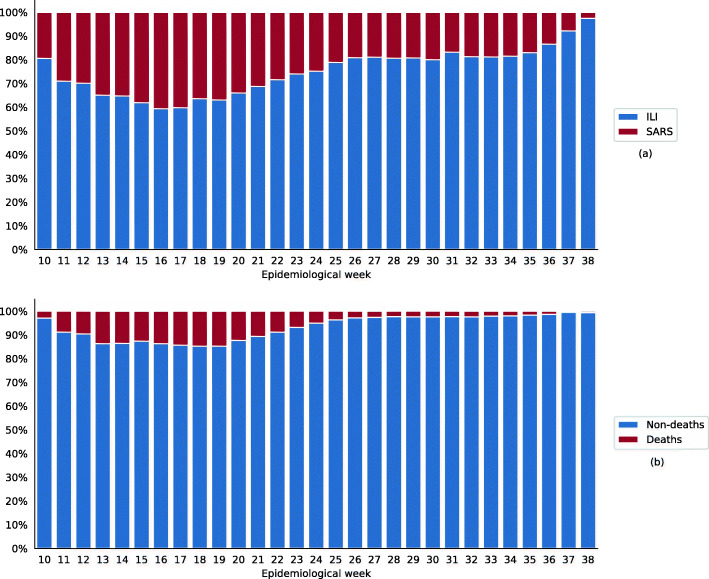
The proportion of cases per epidemiological week according to clinical evolution (ILI/SARS) (**a**) and outcome (**b**). Note: confirmed cases of COVID-19, state of Rio de Janeiro, March–September 2020

The historical case series per epidemiological week showed a proportional increase in cases of ILI relative to those of SARS starting from epidemiological week 19 (Fig. 1a). Figure 1b shows a proportional increase in deaths compared to non-deaths between the 13th and 20th epidemiological weeks, with a maximum of 2465 deaths (14.7%) in week 18. After that, the death percentage dropped.

**Fig. 2:**
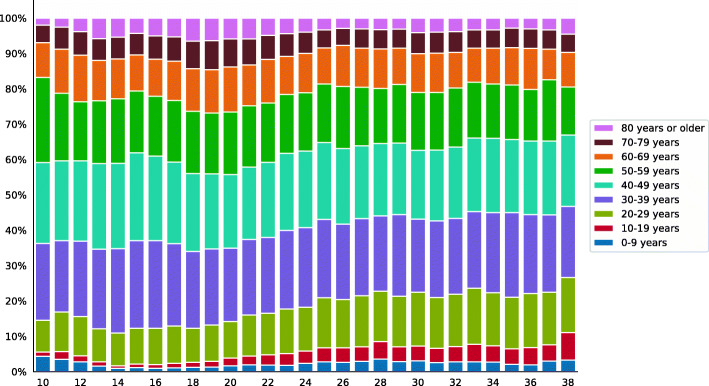
The proportion of cases per epidemiological week according to age group. Note: confirmed cases of COVID-19, state of Rio de Janeiro, March–September 2020

Concomitantly, there was a progressive increase in cases in younger age groups throughout the study period (Fig. 2).

**Fig. 3:**
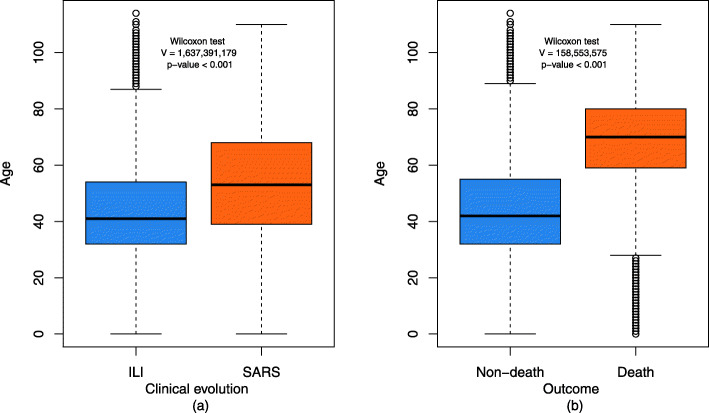
Age distribution according to clinical evolution (ILI/SARS) (**a**) and outcome (**b**). Note: confirmed cases of COVID-19, state of Rio de Janeiro, March–September 2020

Figure 3 illustrates the comparison of the age distribution according to clinical evolution - ILI vs. SARS (a) and death vs. non-death (b). ILI was more frequent in younger individuals and SARS in older individuals. The comparison between the age groups of individuals according to the outcome showed a higher concentration of elderly individuals in the group that progressed to death. In both cases, there was a statistically significant difference.

The geographical distribution of confirmed cases and deaths per municipality of residence is shown in Table 5. The incidence for the entire state was equal to 1522.9 cases per 100,000 inhabitants, the mortality rate was 112.9 deaths per 100,000 inhabitants, and the lethality rate was 7.4%. We observed the highest incidences in Varre-Sai, Macaé, and Natividade, with rates of 6321.9, 3799.7, and 3626.8 cases per 100,000 inhabitants, respectively. The municipalities with the highest lethality rates were Nilópolis, São João do Meriti, and Rio de Janeiro, with 14.3, 13.3, and 11.9%, respectively. Rio de Janeiro, Iguaba Grande, and Tanguá recorded the highest mortality rates, with 170.7, 148.8, and 136.7 deaths per 100,000 inhabitants, respectively.

**Table 5:** Geographic distribution of cases by regions and municipalities of the state of Rio de Janeiro

Region/Municipality	Population	Death	Non-death	Cases	Lethality	Mortality (100 thousand inhabitants)	Incidence (100 thousand inhabitants)
**Central-South Fluminense Region**	**272,407**	**157**	**5620**	**5777**	**2.7%**	**57.6**	**2120.7**
Areal	11,423	6	180	186	3.2%	52.5	1628.3
Comendador Levy Gasparian	8180	4	149	153	2.6%	48.9	1870.4
Engenheiro Paulo de Frontin	13,237	4	135	139	2.9%	30.2	1050.1
Mendes	17,935	3	173	176	1.7%	16.7	981.3
Miguel Pereira	24,642	12	241	253	4.7%	48.7	1026.7
Paraíba do Sul	41,084	21	869	890	2.4%	51.1	2166.3
Paty do Alferes	26,539	7	167	174	4.0%	26.4	655.6
Sapucaia	17,525	22	373	395	5.6%	125.5	2253.9
Três Rios	77,432	59	2624	2683	2.2%	76.2	3465.0
Vassouras	34,410	19	709	728	2.6%	55.2	2115.7
**Costa Verde Region**	**243,500**	**222**	**6694**	**6916**	**3.2%**	**91.2**	**2840.2**
Angra dos Reis	169,511	150	5170	5320	2.8%	88.5	3138.4
Mangaratiba	36,456	48	699	747	6.4%	131.7	2049.0
Paraty	37,533	24	825	849	2.8%	63.9	2262.0
**Coastal Lowlands Region**	**810,666**	**577**	**12,126**	**12,703**	**4.5%**	**71.2**	**1567.0**
Araruama	112,008	63	1224	1287	4.9%	56.2	1149.0
Armação dos Búzios	27,560	15	423	438	3.4%	54.4	1589.3
Arraial do Cabo	27,715	6	184	190	3.2%	21.6	685.5
Cabo Frio	186,227	139	2222	2361	5.9%	74.6	1267.8
Cachoeiras de Macacu	54,273	34	558	592	5.7%	62.6	1090.8
Casimiro de Abreu	35,347	19	898	917	2.1%	53.8	2594.3
Iguaba Grande	22,851	34	576	610	5.6%	148.8	2669.5
Rio Bonito	55,551	55	1805	1860	3.0%	99.0	3348.3
Rio das Ostras	105,676	101	1875	1976	5.1%	95.6	1869.9
São Pedro da Aldeia	87,875	42	1201	1243	3.4%	47.8	1414.5
Saquarema	74,234	56	1010	1066	5.3%	75.4	1436.0
Silva Jardim	21,349	13	150	163	8.0%	60.9	763.5
**Middle Paraíba Region**	**855,193**	**480**	**13,998**	**14,478**	**3.3%**	**56.1**	**1693.0**
Barra do Pirai	94,778	51	1023	1074	4.7%	53.8	1133.2
Barra Mansa	177,813	117	2210	2327	5.0%	65.8	1308.7
Itatiaia	28,783	6	351	357	1.7%	20.8	1240.3
Pinheiral	22,719	10	462	472	2.1%	44.0	2077.6
Pirai	26,314	16	648	664	2.4%	60.8	2523.4
Porto Real	16,592	11	355	366	3.0%	66.3	2205.9
Quatis	12,793	1	144	145	0.7%	7.8	1133.4
Resende	119,769	56	1932	1988	2.8%	46.8	1659.9
Rio Claro	17,425	5	280	285	1.8%	28.7	1635.6
Rio das Flores	8561	2	21	23	8.7%	23.4	268.7
Valença	71,843	17	566	583	2.9%	23.7	811.5
Volta Redonda	257,803	188	6006	6194	3.0%	72.9	2402.6
**Metropolitan Region I**	**9,920,734**	**13,793**	**117,483**	**131,276**	**10.5%**	**139.0**	**1323.2**
Belford Roxo	469,332	292	8546	8838	3.3%	62.2	1883.1
Duque de Caxias	855,048	749	8320	9069	8.3%	87.6	1060.6
Itaguaí	109,091	116	2164	2280	5.1%	106.3	2090.0
Japeri	95,492	37	397	434	8.5%	38.7	454.5
Magé	227,322	236	3474	3710	6.4%	103.8	1632.0
Mesquita	168,376	168	1344	1512	11.1%	99.8	898.0
Nilópolis	157,425	188	1124	1312	14.3%	119.4	833.4
Nova Iguaçu	796,257	603	5564	6167	9.8%	75.7	774.5
Paracambi	47,124	39	677	716	5.4%	82.8	1519.4
Queimados	137,962	83	2341	2424	3.4%	60.2	1757.0
Rio de Janeiro	6,320,446	10,787	79,981	90,768	11.9%	170.7	1436.1
São João de Meriti	458,673	442	2876	3318	13.3%	96.4	723.4
Seropédica	78,186	53	675	728	7.3%	67.8	931.1
**Metropolitan Region II**	**1,914,974**	**1668**	**33,696**	**35,364**	**4.7%**	**87.1**	**1846.7**
Guapimirim	51,483	50	1608	1658	3.0%	97.1	3220.5
Itaboraí	218,008	225	4313	4538	5.0%	103.2	2081.6
Maricá	127,461	134	3391	3525	3.8%	105.1	2765.6
Niterói	487,562	486	12,354	12,840	3.8%	99.7	2633.5
São Gonçalo	999,728	731	11,248	11,979	6.1%	73.1	1198.2
Tanguá	30,732	42	782	824	5.1%	136.7	2681.2
**Northeast Fluminense Region**	**317,493**	**160**	**8070**	**8230**	**1.9%**	**50.4**	**2592.2**
Aperibé	10,213	5	188	193	2.6%	49.0	1889.7
Bom Jesus do Itabapoana	35,411	10	736	746	1.3%	28.2	2106.7
Cambuci	14,827	2	181	183	1.1%	13.5	1234.2
Italva	14,063	7	348	355	2.0%	49.8	2524.4
Itaocara	22,899	17	333	350	4.9%	74.2	1528.5
Itaperuna	95,841	77	2671	2748	2.8%	80.3	2867.2
Laje do Muriaé	7487	3	242	245	1.2%	40.1	3272.3
Miracema	26,843	2	429	431	0.5%	7.5	1605.6
Natividade	15,082	3	544	547	0.5%	19.9	3626.8
Porciúncula	17,760	12	593	605	2.0%	67.6	3406.5
Santo Antônio de Pádua	40,589	19	1068	1087	1.7%	46.8	2678.1
São Jose de Ubá	7003	1	140	141	0.7%	14.3	2013.4
Varre-Sai	9475	2	597	599	0.3%	21.1	6321.9
**North Fluminense Region**	**849,515**	**566**	**15,995**	**16,561**	**3.4%**	**66.6**	**1949.5**
Campos dos Goytacazes	463,731	321	4941	5262	6.1%	69.2	1134.7
Carapebus	13,359	7	278	285	2.5%	52.4	2133.4
Cardoso Moreira	12,600	4	348	352	1.1%	31.7	2793.7
Conceição de Macabu	21,211	10	593	603	1.7%	47.1	2842.9
Macaé	206,728	152	7703	7855	1.9%	73.5	3799.7
Quissamã	20,242	8	445	453	1.8%	39.5	2237.9
São Fidelis	37,543	25	234	259	9.7%	66.6	689.9
São Francisco de Itabapoana	41,354	21	539	560	3.8%	50.8	1354.2
São João da Barra	32,747	18	914	932	1.9%	55.0	2846.1
**Mountain Region**	**805,627**	**433**	**11,498**	**11,931**	**3.6%**	**53.7**	**1481.0**
Bom Jardim	25,333	5	156	161	3.1%	19.7	635.5
Cantagalo	19,830	2	151	153	1.3%	10.1	771.6
Carmo	17,434	3	162	165	1.8%	17.2	946.4
Cordeiro	20,430	7	217	224	3.1%	34.3	1096.4
Duas Barras	10,930	3	62	65	4.6%	27.4	594.7
Macuco	5269	2	65	67	3.0%	38.0	1271.6
Nova Friburgo	182,082	86	2749	2835	3.0%	47.2	1557.0
Petrópolis	295,917	174	2135	2309	7.5%	58.8	780.3
Santa Maria Madalena	10,321	3	103	106	2.8%	29.1	1027.0
São Jose do Vale do Rio Preto	20,251	16	439	455	3.5%	79.0	2246.8
São Sebastiao do Alto	8895	1	30	31	3.2%	11.2	348.5
Sumidouro	14,900	11	139	150	7.3%	73.8	1006.7
Teresópolis	163,746	120	5034	5154	2.3%	73.3	3147.6
Trajano de Moraes	10,289	0	56	56	0.0%	0.0	544.3
Missing	–	20	253	273	7.3%	–	–
**Total**	**15,990,109**	**18,076**	**225,433**	**243,509**	**7.4%**	**112.9**	**1522.9**

Note: confirmed cases of COVID-19, state of Rio de Janeiro, March–September 2020

Regarding the regions of the state of Rio de Janeiro, Metropolitan Region I had the highest lethality (10.5%) and mortality (139.0 deaths per 100,000 inhabitants), while the highest incidence was in the Costa Verde Region (2840.2 cases per 100,000 inhabitants).

**Fig. 4:**
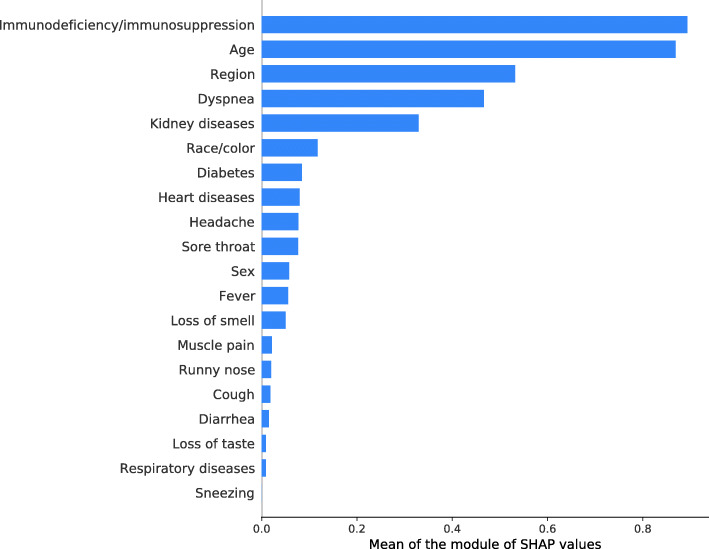
Means of the SHAP metric of the predictor variables of the XGBoost model. Note: confirmed cases of COVID-19, state of Rio de Janeiro, March–September 2020

The values in Fig. 4 illustrate the mean values of the SHAP metric of the most relevant predictor variables in defining the odds of death of the XGBoost model. Immunodeficiency/immunosuppression, age, region of residence, and presence of dyspnea were the characteristics with the most significant impact on the outcome.

Table 6 shows the evaluation measures of the XGBoost model. Its high sensitivity (89%) and specificity (89%) indicate the model’s ability to identify cases of death and non-death, respectively. The accuracy revealed 89% of hits, while the negative predictive value showed that 99% of the cases predicted as non-deaths were correct. The positive predictive value of 30% may reflect the data imbalance since more than 90% of the cases had a non-death outcome.

**Table 6:** Evaluation measures of the XGBoost model

**Sensitivity**	89%
**Specificity**	89%
**Accuracy**	89%
**Youden’s index**	0.78
**The area under the ROC curve**	94%
**Positive predictive value**	30%
**Negative predictive value**	99%
**MCC**	48%

Note: confirmed cases of COVID-19, state of Rio de Janeiro, March–September 2020

**Fig. 5:**
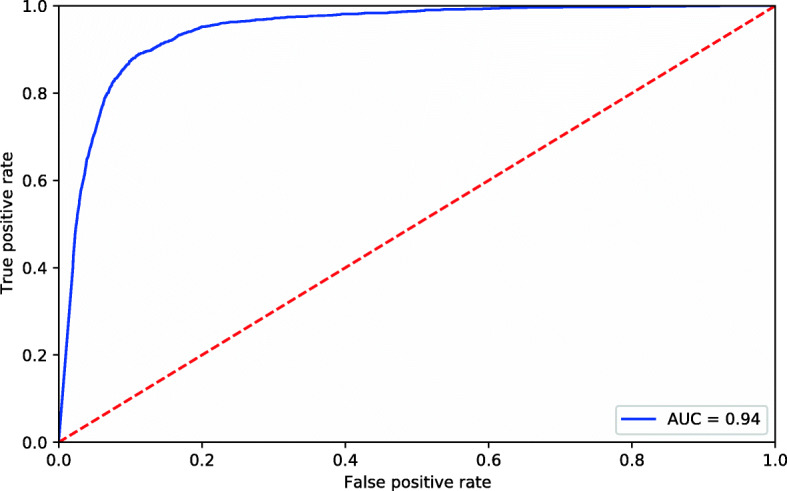
Receiver operating characteristic (ROC) curve of the XGBoost model. Note: confirmed cases of COVID-19, state of Rio de Janeiro, March–September 2020

Figure 5 shows the ROC curve, which appears very close to the upper left corner, with an area under the curve of 94%, revealing that the model performed excellently in predicting the outcome.

Table 7 shows the odds ratio of death with the respective confidence intervals of the predictor variables. Note that the variables with the highest odds of death were metropolitan region I - capital, metropolitan region I - without capital, and metropolitan region II (compared to the northwest fluminense region); the presence of dyspnea, presence of fever, presence of diabetes, presence of heart disease (compared to absence); black race/color (compared to white color/race); male sex (compared to female); and age over 30 years (compared to age range 0 to 9 years). The variables associated with lower odds of death were coastal lowlands region (compared to the northwest fluminense region); the presence of headache, presence of odynophagia, presence of muscle pain, presence of diarrhea, presence of loss of smell, and presence of runny nose (compared to absence); and Asian race/color (compared to white color/race).

**Table 7:** Odds ratios and respective 95% confidence intervals were obtained from the results of the *XGBoost* model

Predictor variable and reference category	Category of interest	Odds ratio (95% CI)	***P***-value
**Sex (reference: female)**	Male	1.029 (1.012; 1.046)	0,001
**Race/color (reference: white)**	Asian	0.935 (0.899; 0.973)	0,001
	Indigenous	1.029 (0.857; 1.236)	0,772
	Brown	1.032 (0.996; 1.069)	0,081
	Black	1.033 (1.012; 1.054)	0,002
	Missing	1.078 (1.054; 1.102)	0,000
**Respiratory diseases (reference: absence)**	Presence	0.982 (0.932; 1.033)	0,499
	Missing	0.657 (0.539; 0.800)	0,000
**Kidney diseases (reference: absence)**	Presence	1.011 (0.928; 1.102)	0,815
	Missing	1.336 (1.300; 1.373)	0,000
**Diabetes (reference: absence)**	Presence	1.176 (1.137; 1.218)	0,000
	Missing	1.021 (0.992; 1.051)	0,159
**Heart diseases (reference: absence)**	Presence	1.073 (1.045; 1.103)	0,000
	Missing	1.006 (0.975; 1.037)	0,717
**Immunodeficiency/Immunosuppression (reference: absence)**	Presence	1.007 (0.930; 1.091)	0,874
	Missing	1.685 (1.639; 1.732)	0,000
**Age (reference: 0 to 9 years)**	10–19 years	1.004 (0.934; 1.078)	0,920
	20–29 years	1.011 (0.953; 1.073)	0,731
	30–39 years	1.061 (1.001; 1.124)	0,045
	40–49 years	1.116 (1.053; 1.183)	0,000
	50–59 years	1.285 (1.212; 1.363)	0,000
	60–69 years	1.585 (1.491; 1.684)	0,000
	70–79 years	1.811 (1.697; 1.933)	0,000
	80 years or older	1.869 (1.744; 2.002)	0,000
**Dyspnea (reference: absence)**	Presence	1.309 (1.283; 1.336)	0,000
	Missing	1.411 (1.313; 1.516)	0,000
**Cough (reference: absence)**	Presence	1.006 (0.989; 1.022)	0,485
	Missing	1.411 (1.313; 1.516)	0,000
**Fever (reference: absence)**	Presence	1.029 (1.011; 1.046)	0,001
	Missing	1.019 (0.951; 1.092)	0,606
**Headache (reference: absence)**	Presence	0.909 (0.888; 0.931)	0,000
	Missing	0.976 (0.911; 1.045)	0,498
**Sneezing (reference: absence)**	Presence	1.000 (0.905; 1.106)	1.000
	Missing	1.000 (0.934; 1.071)	1.000
**Odynophagia (reference: absence)**	Presence	0.933 (0.913; 0.954)	0,000
	Missing	0.985 (0.919; 1.055)	0,681
**Muscle pain (reference: absence)**	Presence	0.964 (0.938; 0.992)	0,010
	Missing	0.985 (0.919; 1.055)	0,681
**Diarrhea (reference: absence)**	Presence	0.954 (0.920; 0.989)	0,011
	Missing	0.990 (0.924; 1.060)	0,787
**Loss of smell (reference: absence)**	Presence	0.913 (0.887; 0.939)	0,000
	Missing	0.959 (0.896; 1.027)	0,231
**Loss of taste (reference: absence)**	Presence	0.976 (0.944; 1.010)	0,159
	Missing	0.989 (0.923; 1.059)	0,765
**Runny nose (reference: absence)**	Presence	0.955 (0.926; 0.985)	0,004
	Missing	0.983 (0.918; 1.053)	0,637
**Region of residence (reference: Northwest Fluminense)**	Central-South Fluminense Region	0.955 (0.893; 1.023)	0,185
	Costa Verde Region	0.941 (0.883; 1.004)	0,063
	Coastal Lowlands Region	0.944 (0.893; 0.999)	0,044
	Middle Paraíba Region	1.000 (0.946; 1.056)	1000
	Metropolitan Region I - Capital	1.322 (1.263; 1.384)	0,000
	Metropolitan Region I - Without Capital	1.269 (1.209; 1.332)	0,000
	Metropolitan Region II	1.113 (1.060; 1.169)	0,000
	North Fluminense Region	0.984 (0.933; 1.038)	0,565
	Mountain Region	0.962 (0.909; 1.018)	0,181
	Missing	0.942 (0.739; 1.201)	0,642

Note: confirmed cases of COVID-19, state of Rio de Janeiro, March–September 2020

## Discussion

In the present study, the mortality and lethality of COVID-19 increased with age, showing that the disease tends to be more severe in the older population. The Chinese Center for Disease Control and Prevention [31] analyzed 44,672 confirmed cases of the disease and found similar findings. However, the Chinese study found much lower lethality in all age groups than in this study. In the age group of 70 to 79 years, the lethality of the present study was 3.6 times higher than the lethality in the Chinese study (28.97% versus 8.0%), while in the age group of 80 years and over, the lethality was 2.9 times higher (42.75% versus 14.8%).

Older people tend to have lower immunity and, therefore, greater susceptibility to severe forms of infectious diseases and a higher frequency of comorbidities such as cardiovascular diseases, diabetes, and chronic lung diseases. These factors may explain the higher frequency of severe form (SARS) cases in addition to higher lethality among people over 60 years. Advanced age as a risk factor for death has been described in Noor et al. [18] and Albitar et al. [19].

The higher incidence of COVID-19 in the age group between 30 and 49 years was probably attributable to their greater exposure to SARS-CoV-2 in public transport or at work since the need to generate income prevented many people from staying on lockdown. Conversely, the evolution of the frequency of cases of SARS and ILI over time showed a proportional increase in ILI relative to SARS, starting from epidemiological week 19. Over the same time, we observed an increase in the frequency of cases in younger age groups, which may be related to lower adherence to social distancing in this population group. There was a higher frequency of female patients in this study. Williamson et al. [16] Cobre et al. [17] found similar results. We show the association of black race/skin color with higher odds of death than white race/skin color. Williamson et al. [16] found a higher risk of death in black individuals than in white individuals, even after adjusting for other factors (hazard ratio 1.48 (95% CI 1.29–1.69)).

Dyspnea, cough, and fever were the most commonly found symptoms, being more frequent in SARS cases and cases of death. Fever is a clinical sign that is part of the set of organic inflammatory responses to the presence of an infectious agent and is mediated mainly by cytokines. These inflammatory mediators can cause tissue damage and organ dysfunction by stimulating free radicals and other toxic components, as cited in the study by Chang et al. [32]. The latter retrospectively followed 211 patients and investigated risk factors for the progression of COVID-19. Therefore, fever may be a clinical sign associated with greater disease severity. Dyspnea, in turn, is the main symptom of SARS, a severe form of the disease where more deaths are expected. Fever and dyspnea were associated with a higher chance of death. At the same time, other reported symptoms, such as headache, odynophagia, muscle pain, diarrhea, loss of smell, and runny nose, had an association with a lower chance of death. Maciel et al. reported dyspnea as a factor associated with COVID-19 death, while cough, fever, and other symptoms were protective factors [33].

The city of Rio de Janeiro had the highest mortality within the state. When mortality is standardized, the Standardized Mortality Ratio (SMR) of the city of Rio de Janeiro remained the highest (supplementary Table 1). On the other hand, lethality due to the main risk factors identified in this study showed that lethality was consistently higher in the city of Rio de Janeiro (supplementary Table 2). However, the prevalence of risk factors among cases of COVID-19 does not change significantly between regions (Supplementary Figure A). In addition, the city of Rio de Janeiro has significant social inequality, with about 22% of the population residing in socially vulnerable places called favelas (supplementary Table 3). This combination of factors may have contributed to the high mortality and lethality in the city of Rio de Janeiro. It is also noteworthy that the city of Rio de Janeiro has a better laboratory diagnostic capacity for COVID-19, which would lead to a more accurate classification of the underlying cause of death than in other regions of the state of Rio de Janeiro. Also, there is better access of the population to health services in the city, facilitating the confirmation of the diagnosis on time. The difficulty of laboratory diagnosis of COVID-19 and access to health services in most cities of the state may have contributed to inaccuracy in the diagnosis of COVID-19 [34]. A less robust health system, especially when associated with a lower socioeconomic level, seems to be a risk factor for higher lethality due to COVID-19 [20].

In this study, people with heart disease had a higher chance of death. Zheng et al. [34] described the association of some comorbidities with higher lethality from COVID-19 in a meta-analysis. Cardiac tissue has a higher number of angiotensin-converting enzyme receptors (ACE2), which are involved in the endocytosis of SARS-CoV-2, than other tissues, which could allow the virus to damage cardiac tissue directly. Mikami et al. analyzed 6493 hospitalized patients with a confirmed diagnosis of COVID-19 and found a higher risk of death in patients with elevated serum troponin levels [35].

This study found a higher chance of death in people with diabetes. Hyperglycemia secondary to diabetes mellitus can lead to immune dysfunction through the impairment of humoral and cellular functions and the antioxidant system. Besides, diabetic patients are more vulnerable to nosocomial infections [36]. These factors may be associated with a higher chance of death in diabetic patients with COVID-19.

Prognostic factors identified in the literature include laboratory and radiological findings [37, 38], which were not analyzed in this study due to the lack of information in the databases that were used. It is important to highlight that bad prognostic indicators do not guarantee an unfavorable evolution of the disease [39].

Some limitations of our study should be noted. The *RedCap* platform has received input of ILI notifications until March 27, 2020, and the e-SUS Notifica has received input of ILI notifications after that. The notifications of SARS are made by sending the scanned investigation form, which feeds the Influenza Epidemiological Surveillance Information System (SIVEP-*Gripe*). In this context, the reported cases refer to those who sought care in health units in the state of Rio de Janeiro and entered into these information systems. The instructions given to the population were to seek medical care in case of signs of severity, which might have generated a selection bias.

Another factor that should be taken into account is the missing data for the variables skin color/race, chronic kidney disease, immunodeficiency/immunosuppression, which may have generated information bias.

It is also important to mention that asymptomatic cases are not included in this study, which can lead to an overestimation of the lethality rate.

At the time of this study, there was no consensus of management of COVID-19 in Brazil. Thus, several health services have adopted their own protocols. There was no single protocol for all municipalities in the state of Rio de Janeiro. It was a very important feature of the Brazilian management of the pandemic and negatively affected the observed results.

The authors recognize the importance of risk factors such as smoking, chronic hepatic disease, and cancer in the analysis of COVID-19, but they were not present in the available databases.

## Conclusions

COVID-19 is a disease that can evolve into severe forms leading to death, especially in certain population groups. This study showed that older individuals of black race/skin color with heart disease or diabetes who had dyspnea or fever were more likely to die. The present study aimed to contribute to the early identification of COVID-19 patients who may progress to a more severe form of the disease, improve the clinical management of patients with COVID-19, and reduce the disease’s lethality.

## Supplementary Information

**Additional file 1.**

## Data Availability

The databases and materials used in this manuscript may be made available upon request from interested researchers. To that end, please contact the corresponding author, Marcella Cini Oliveira, through the e-mail cini.marcella@gmail.com.

## Abbreviations

ACE2: Angiotensin-converting enzyme receptors
COVID-19: Coronavirus disease 2019
ENN: Edited nearest neighbor
XGBoost: Extreme gradient boosting
ILI: Influenza-like illness
MCC: Matthews’ correlation coefficient
ROC: Receiver operating characteristic
RT-PCR: Reverse transcription polymerase chain reaction
SARS: Severe acute respiratory syndrome
SARS-CoV-2: Severe acute respiratory syndrome coronavirus 2
SHAP: SHapley Additive exPlanations
SIVEP-Gripe: Flu Epidemiological Surveillance Information System
SMOTE: Synthetic minority oversampling technique
SMR: Standardized Mortality Ratio
SUS: Unified Health System
WHO: World Health Organization
