# Correction to: Factors associated with death in confirmed cases of COVID-19 in the state of Rio de Janeiro

**DOI:** 10.1186/s12879-021-06410-2

**Published:** 2021-08-02

**Authors:** Marcella Cini Oliveira, Tatiana de Araujo Eleuterio, Allan Bruno de Andrade Corrêa, Lucas Dalsenter Romano da Silva, Renata Coelho Rodrigues, Bruna Andrade de Oliveira, Marlos Melo Martins, Carlos Eduardo Raymundo, Roberto de Andrade Medronho

**Affiliations:** 1 Faculdade de Medicina, Universidade Federal do Rio de Janeiro, Rio de Janeiro, Brazil; 2 Instituto de Estudos em Saúde Pública / Faculdade de Medicina, Universidade Federal do Rio de Janeiro, Rio de Janeiro, Brazil; 3 Faculdade de Enfermagem, Universidade do Estado do Rio de Janeiro, Rio de Janeiro, Brazil; 4 Instituto de Física, Universidade Federal do Rio de Janeiro, Rio de Janeiro, Brazil; 5 Departamento de Medicina Preventiva, Instituto de Estudos em Saúde Pública / Faculdade de Medicina, Universidade Federal do Rio de Janeiro, Rio de Janeiro, Brazil; 6 Department of Child Neurology, Martagão Gesteira Institute of Childcare and Pediatrics, Federal University of Rio de Janeiro, Rio de Janeiro, Brazil

**Correction to: BMC Infect Dis 21, 687 (2021)**

**https://doi.org/10.1186/s12879-021-06384-1**

Following publication of the original article [1], the authors identified an error in the article title.

The incorrect title is:

Fatores associados ao óbito em casos confirmados de COVID-19 no estado do Rio de JaneiroThe

The correct title is:

Factors associated with death in confirmed cases of COVID-19 in the state of Rio de Janeiro

The original article [1] has been corrected.
