# Supplementary material for: Fatores associados ao óbito em casos confirmados de COVID-19 no estado do Rio de Janeiro
Source: BMC Infect Dis. 2021 Jul 16;21:687. doi: 10.1186/s12879-021-06384-1 (PMC8283387; doi:10.1186/s12879-021-06384-1)
Supplement: Supplementary file 1 — Additional file 1. [file 12879_2021_6384_MOESM1_ESM.docx]

**Reviewer comment:**

**Discussion, para5, the authors state that the region was listed as the third risk of death risk of COVID-19, and the underlying cause was the capability of the COVID-19 test. There is extraordinarily high in lethality and mortality in the metropolitan region I while a similar incidence compared to other regions. Are there any other concerns contributing to the highest lethality and mortality? Mutated SARS-CoV-2? Overlaps in those risk factors mentioned by the authors? heterogeneity in population composition? A brief organized discussion is suggested.**

**Authors response:**

The city of Rio de Janeiro had the highest mortality within the state. When mortality is standardized, the Standardized Mortality Ratio (SMR) of the city of Rio de Janeiro remained the highest (supplementary table 1). On the other hand, lethality due to the main risk factors identified in this study showed that lethality was consistently higher in the city of Rio de Janeiro (supplementary table 2). However, the prevalence of risk factors among cases of COVID-19 does not change significantly between regions (supplementary figure A). In addition, the city of Rio de Janeiro has significant social inequality, with about 22% of the population residing in socially vulnerable places called favelas (supplementary table 3). This combination of factors may have contributed to the high mortality and lethality in the city of Rio de Janeiro. It is also noteworthy that the city of Rio de Janeiro has a better laboratory diagnostic capacity for COVID-19, which would lead to a more accurate classification of the underlying cause of death than in other regions of the state of Rio de Janeiro. Also, there is better access of the population to health services in the city, facilitating the confirmation of the diagnosis on time. The difficulty of laboratory diagnosis of COVID-19 and access to health services in most cities of the state may have contributed to inaccuracy in the diagnosis of COVID-19 (35). A less robust health system, especially when associated with a lower socioeconomic level, seems to be a risk factor for higher lethality due to COVID-19 (21).

**Supplementary table 1. Standardized Mortality Ratio (SMR) by region in cases of COVID-19.**

| **Region/Municipality** | **SMR** |
| --- | --- |
| **Central-South Fluminense Region** | 44,0 |
| **Costa Verde Region** | 77,9 |
| **Coastal Lowlands Region** | 45,9 |
| **Middle Paraíba Region** | 45,6 |
| **Metropolitan Region I - Capital** | 102,9 |
| **Metropolitan Region I - without Capital** | 66,4 |
| **Metropolitan Region II** | 57,8 |
| **Northeast Fluminense Region** | 34,8 |
| **North Fluminense Region** | 56,6 |
| **Mountain Region** | 47,3 |

**Supplementary table 2. Outcome of confirmed cases of COVID-19 by Region and risk fator.**

| **Region** | **Risk factor** | **Outcome** | | | |
| --- | --- | --- | --- | --- | --- |
|  |  | **Deaths** | | **Non-deaths** | |
|  |  | **n** | **%** | **n** | **%** |
| **Central-South Fluminense Region** |  |  |  |  |  |
|  | Sex - Male | 94 | 3.5% | 2.595 | 96.5% |
|  | Race/ color - Black | 9 | 1.9% | 476 | 98.1% |
|  | Presence of diabetes | 45 | 14.4% | 267 | 85.6% |
|  | Presence of heart diseases | 55 | 7.1% | 715 | 92.9% |
|  | Age - 30 years or older | 154 | 3.7% | 4.049 | 96.3% |
|  | **Total** | **157** | **2.7%** | **5.620** | **97.3%** |
| **Costa Verde Region** |  |  |  |  |  |
|  | Sex - Male | 122 | 3.7% | 3.210 | 96.3% |
|  | Race/ color - Black | 16 | 5.9% | 255 | 94.1% |
|  | Presence of diabetes | 55 | 15.2% | 308 | 84.8% |
|  | Presence of heart diseases | 50 | 9.5% | 477 | 90.5% |
|  | Age - 30 years or older | 216 | 4.0% | 5.156 | 96.0% |
|  | **Total** | **222** | **3.2%** | **6.694** | **96.8%** |
| **Coastal Lowlands Region** |  |  |  |  |  |
|  | Sex - Male | 327 | 5.3% | 5.821 | 94.7% |
|  | Race/ color - Black | 41 | 4.6% | 857 | 95.4% |
|  | Presence of diabetes | 142 | 17.9% | 653 | 82.1% |
|  | Presence of heart diseases | 167 | 13.5% | 1.069 | 86.5% |
|  | Age - 30 years or older | 561 | 5.4% | 9.795 | 94.6% |
|  | **Total** | **577** | **4.5%** | **12.126** | **95.5%** |
| **Middle Paraíba Region** |  |  |  |  |  |
|  | Sex – Male | 283 | 4.2% | 6.462 | 95.8% |
|  | Race/ color - Black | 59 | 4.9% | 1.152 | 95.1% |
|  | Presence of diabetes | 111 | 15.0% | 629 | 85.0% |
|  | Presence of heart diseases | 140 | 13.5% | 895 | 86.5% |
|  | Age - 30 years or older | 469 | 4.2% | 10.764 | 95.8% |
|  | **Total** | **480** | **3.3%** | **13.998** | **96.7%** |
| **Metropolitan Region I – Capital** |  |  |  |  |  |
|  | Sex – Male | 6.000 | 14.0% | 36.981 | 86.0% |
|  | Race/ color - Black | 876 | 15.7% | 4.719 | 84.3% |
|  | Presence of diabetes | 2.517 | 36.7% | 4.344 | 63.3% |
|  | Presence of heart diseases | 3.691 | 33.6% | 7.288 | 66.4% |
|  | Age - 30 years or older | 10.608 | 13.6% | 67.545 | 86.4% |
|  | **Total** | **10.787** | **11.9%** | **79.981** | **88.1%** |
| **Metropolitan Region I - Without Capital** |  |  |  |  |  |
|  | Sex – Male | 1.650 | 8.8% | 17.088 | 91.2% |
|  | Race/ color - Black | 249 | 10.4% | 2.151 | 89.6% |
|  | Presence of diabetes | 652 | 25.9% | 1.866 | 74.1% |
|  | Presence of heart diseases | 866 | 20.0% | 3.457 | 80.0% |
|  | Age - 30 years or older | 2.919 | 8.9% | 29.825 | 91.1% |
|  | **Total** | **3.006** | **7.4%** | **37.502** | **92.6%** |
| **Metropolitan Region II** |  |  |  |  |  |
|  | Sex – Male | 942 | 5.6% | 16.030 | 94.4% |
|  | Race/ color - Black | 120 | 5.3% | 2.133 | 94.7% |
|  | Presence of diabetes | 459 | 20.9% | 1.732 | 79.1% |
|  | Presence of heart diseases | 699 | 18.1% | 3.160 | 81.9% |
|  | Age - 30 years or older | 1.641 | 5.7% | 27.095 | 94.3% |
|  | **Total** | **1.668** | **4.7%** | **33.696** | **95.3%** |
| **Northwest Fluminense** |  |  |  |  |  |
|  | Sex – Male | 83 | 2.1% | 3.831 | 97.9% |
|  | Race/ color - Black | 17 | 2.2% | 757 | 97.8% |
|  | Presence of diabetes | 32 | 13.1% | 212 | 86.9% |
|  | Presence of heart diseases | 50 | 9.5% | 475 | 90.5% |
|  | Age - 30 years or older | 153 | 2.5% | 5.954 | 97.5% |
|  | **Total** | **160** | **1.9%** | **8.070** | **98.1%** |
| **North Fluminense Region** |  |  |  |  |  |
|  | Sex – Male | 315 | 3.6% | 8.411 | 96.4% |
|  | Race/ color - Black | 44 | 3.9% | 1.081 | 96.1% |
|  | Presence of diabetes | 193 | 26.0% | 549 | 74.0% |
|  | Presence of heart diseases | 239 | 20.9% | 902 | 79.1% |
|  | Age - 30 years or older | 561 | 4.3% | 12.538 | 95.7% |
|  | **Total** | **566** | **3.4%** | **15.995** | **96.6%** |
| **Mountain Region** |  |  |  |  |  |
|  | Sex – Male | 247 | 4.5% | 5.220 | 95.5% |
|  | Race/ color - Black | 46 | 5.9% | 732 | 94.1% |
|  | Presence of diabetes | 132 | 18.2% | 594 | 81.8% |
|  | Presence of heart diseases | 204 | 14.3% | 1.225 | 85.7% |
|  | Age - 30 years or older | 428 | 4.7% | 8.770 | 95.3% |
|  | **Total** | **433** | **3.6%** | **11.498** | **96.4%** |

**Supplementary table 3. The situation of households and urbanism in the city of Rio de Janeiro.**

| **Permanent Private Households _(1)_** | 2,144,445 |
| --- | --- |
| **Number of People per Household _(1)_** | 2.9 |
| **Number of Favelas _(2)_** | 1.018 |
| **Population in Favelas _(3)_** | 1,434,975 inhabitants |
| **Percentage of Population in Favelas _(3)_** | 22 % |

**Data:**(1) - IBGE 2010; (2) - IPP 2016; (3) - IPP 2010 based on the 2010 Census. <https://www.data.rio/pages/rio-em-sntese-2>.

**Supplemmentary figure A. Prevalence of the main risk factors identified among cases of COVID-19 by regions of the state of Rio de Janeiro.**
